# Tumor susceptibility gene 101 is required for the maintenance of uterine epithelial cells during embryo implantation

**DOI:** 10.1186/s12958-021-00788-z

**Published:** 2021-07-16

**Authors:** Hyunji Byun, Sojung Kwon, Kay-Uwe Wagner, Hyejin Shin, Hyunjung Jade Lim

**Affiliations:** 1 Department of Veterinary Medicine, School of Veterinary Medicine, Konkuk University, 120 Neungdong-ro, Gwangjin-gu, Seoul, 05029 Republic of Korea; 2 Department of Oncology, Wayne State University School of Medicine and Tumor Biology Program, Barbara Ann Karmanos Cancer Institute, 4100 John R, EL01TM, Detroit, MI 48201 USA; 3 Herbal Medicine Research Division, Korea Institute of Oriental Medicine, Daejeon, 34054 Republic of Korea

**Keywords:** *Tsg101*, Uterus, Epithelium, Implantation, Necroptosis

## Abstract

**Background:**

The tumor susceptibility gene 101 (Tsg101), a component of the endosomal sorting complex required for transport (ESCRT) complex I, is involved in multiple biological processes involving endomembranous structures and the plasma membrane. The role of *Tsg101* in the uterine epithelium was investigated in *Tsg101* floxed mice crossed with *Lactoferrin*-*iCre* mice (*Tsg101*^*d/d*^).

**Methods:**

*Tsg101*^*d/d*^ mice were bred with stud male mice and the status of pregnancy was examined on days 4 and 6. Histological analyses were performed to examine the uterine architecture. Immunofluorescence staining of several markers was examined by confocal microscopy. Uterine epithelial cells (UECs) were isolated from *Tsg101*^*f/f*^ and *Tsg101*^*d/d*^ mice, and the expression of necroptosis effectors was examined by RT-PCR, western blotting, and immunofluorescence staining. UECs were also subjected to RNA expression profiling.

**Results:**

*Tsg101*^*d/d*^ female mice were subfertile with implantation failure, showing unattached blastocysts on day 6 of pregnancy. Histological and marker analyses revealed that some *Tsg101*^*d/d*^ day 4 pregnant uteri showed a disintegrated uterine epithelial structure. *Tsg101*^*d/d*^ UECs began to degenerate within 18 h of culture. In UECs, expression of necroptosis effectors, such as RIPK1, RIPK3, and MLKL were first confirmed. UECs responded to a stimulus to activate necroptosis and showed increased cell death.

**Conclusions:**

*Tsg101* deficiency in the uterine epithelium causes implantation failure, **which may be caused by epithelial defects**. This study provides evidence that UECs harbor a necroptotic machinery that responds to death-inducing signals. Thus, Tsg101 expression in the uterine epithelium is required for normal pregnancy in mice.

## Background

The endosomal sorting complex required for transport (ESCRT) complexes, ESCRT-0, −I, −II, and -III, act in sequence as key regulators of endosomal sorting and maturation [1]. The ESCRT-I complex contains tumor susceptibility gene 101 (Tsg101), vacuolar protein sorting-associated protein 28 homolog Vsp28, Vsp37, and multivesicular body sorting factor 12 (Mvb12) proteins [1]. As a component of ESCRT-I, Tsg101 forms a complex with other ESCRT factors and is essential for the recruitment of subsequent ESCRT complexes [2]. Tsg101 protein has a ubiquitin-interacting domain and downregulates ubiquitinated cell surface receptors and certain protein aggregates [3, 4]. It is also involved in cytokinesis and viral exit from infected cells and is localized in the membrane severing point during these processes [5, 6].

Tsg101 is recognized as a crucial component of ESCRT complexes, and its deletion generally leads to a severe phenotype of cell death [7]. As this protein is involved in various cellular processes, it is often challenging to investigate the aspect of Tsg101 function that leads to cell death of Tsg101-depleted cells in specific contexts. For example, in mammary epithelial cells and mouse embryonic fibroblasts (MEFs), Tsg101 deletion leads to cell cycle arrest [7, 8]. Tsg101-depleted MEF cells exhibit enlarged lysosomes [8]. In Tsg101-depleted HeLa cells, the formation of multivesicular bodies (MVBs), which are late endosomal structures, is severely compromised [4]. When expressed in cells, Tsg101 is generally observed in intracellular vascular structures [4]. It is now well established that Tsg101 is required for endolysosomal maturation and trafficking [6]. Furthermore, Tsg101 deletion in epithelial monolayers leads to loss of epithelial polarity in canine kidney cells, suggesting that it is required for establishing an epithelial barrier [9]. Systemic deletion of *Tsg101* results in early embryonic death between embryonic days 5.5 and 6.5 due to defective cell proliferation [10].

The role of Tsg101 has further been elucidated with the discovery that ESCRT factors protect cells from membrane damage by counteracting necroptotic cell death [11]. Necroptosis often begins with the activation of death receptors by cognate ligands, such as tumor necrosis factor α (TNFα), TNF related apoptosis-inducing ligand (TRAIL), and FAS ligand (FASL). Intracellular signaling follows involving receptor-interacting protein kinase (RIPK) 1, RIPK3, and mixed lineage kinase-like (MLKL) proteins [12]. In various cell types, RIPK1, RIPK3, and MLKL respond to necroptosis-inducing signals and undergo phosphorylation [12]. In L929 mouse fibroblast cells, combined treatment with TNFα (T), LCL161 (S, a Smac mimetic), and zVAD-fmk (Z, an apoptosis inhibitor) induced the phosphorylation of these three factors [13]. Phosphorylated MLKL (pMLKL) proteins translocate to the plasma membrane and mediate membrane permeabilization [11]. MLKL activation results in Ca^2+^ influx, which is rapidly followed by lipid scrambling of the plasma membrane. The damaged plasma membrane depends on certain ESCRT components to maintain integrity following MLKL activation. Charged multivesicular body protein 4B (CHMP4B) and other ESCRT factors produce small membrane vesicles to mend the plasma membrane during necroptosis [11]. Tsg101 promotes the translocation of ESCRT-III factors to the sites of membrane damage and counteracts plasma membrane rupture during necroptosis [11].

In mice, embryo implantation occurs around midnight on day 4 of pregnancy [14]. For this process to proceed successfully, the luminal epithelium undergoes steroid hormone-induced proliferation and differentiation, which renders it competent for embryo attachment [15]. During early pregnancy, steroid hormone levels fluctuate depending on the day of pregnancy [14]. On day 1 of pregnancy, when preovulatory estrogen is dominant, the uterine epithelium proliferates extensively. On day 4, progesterone levels increase and a small amount of estrogen is secreted, driving epithelial differentiation and stromal proliferation in the uterus. On day 6 of pregnancy, when implantation has already taken place, the primary hormone modulating the uterus is progesterone secreted from the corpora lutea [14]. The communication between an implantation-competent blastocyst and a receptive uterus is central to the implantation process and successful pregnancy, and any defect in this process results in implantation failure [15]. The uterine epithelium at the time of embryo implantation undergoes differentiation, expressing several factors involved in two-way interactions. The importance of epithelial polarity in embryo implantation has been demonstrated in a study examining the role of planar cell polarity signaling [16].

*Lactoferrin* (*Ltf*) encodes a non-heme iron-binding glycoprotein and is highly responsive to estrogen in the mouse uterus [17]. *Ltf* is expressed in the uterine epithelium of adult mice but not in immature mouse uteri [18]. A mouse *Cre* model taking advantage of this expression pattern is available as *Ltf-iCre* knock-in mice [19], in which *iCre* is expressed under the endogenous *Ltf* promoter. This *Cre* model efficiently recombines the floxed target gene, primarily in the uterine epithelium, in adult female mice and immature females after estrogen treatment [19]. In this study, we generated a uterine epithelium-specific *Tsg101* deletion model by crossing *Tsg101* floxed (*Tsg101*^*f/f*^) mice with *Ltf-iCre* mice to examine its role in this cell type. Our results show that Tsg101 is required for the maintenance of the uterine epithelium, and its deletion may cause disintegration of the uterine epithelial layer, which may lead to compromised implantation.

## Materials and methods

### Reagents

17β-estradiol (E_2_) (Sigma-Aldrich, St. Louis, MO, USA) was dissolved in sesame oil (Acros Organics). Equine chorionic gonadotropin (eCG) and human chorionic gonadotropin (hCG) were purchased from Sigma-Aldrich.

### Mice

All mice were maintained in accordance with the policies of the Konkuk University International Animal Care and Use Committee (IACUC). *Tsg101* floxed mice (*Tsg101*^*f/f*^) mice [10] were crossed with *Ltf-iCre* mice [19] to obtain *Ltf-iCre*; *Tsg101*^*f/f*^ (*Tsg101*^*d/d*^) mice. *Tsg101*^*d/d*^ mice were produced by crossing *Tsg101*^*f/f*^ female mice with *Ltf-iCre*; *Tsg101*^*f/d*^ male or *Ltf-iCre*; *Tsg101*^*f/f*^ male mice. Genomic DNA was extracted from mouse tails using Gentra Puregene Mouse Tail kit (Qiagen, Hilden, Germany). Genotyping PCR for the floxed *Tsg101* and *Ltf-iCre* genes was performed using the primers shown in Table 1. This study was approved by the Konkuk University IACUC (approval number KU20036).

**Table 1:** Primers used for genotyping and RT-PCR analyses

Gene	Sequence (5′-3′)	Annealing temperature (°C)	No. of cycles	Product size (bp)
*Tsg101* wildtype	F: CCG TGA TCT CTT GAT TCT TCT CC R: CCT GCT CTT TAC TGA AGG CTC	58	35	482
*Tsg101* floxed	F: CCG TGA TCT CTT GAT TCT TCT CC R: GAA ATC CAC CTG CCT CTG CCT C	58	35	482
*Ltf*^*iCre*^ transgene	F: AAC TAG CAC ACC TGG TTG AGG R: CAG GTT TTG GTG CAC AGT CA	60.5	10	215
*Des*	F: CAA AGG GGT TCT GAA GTC CA R: GAA AAG TGG CTG GGT GTG AT	59	28	198
*Krt12*	F: GTC TCA TCC CAG GTT CAG GA R: TGC AAT GAA GAC CAG CAG AG	59	26	231
*Rpl7*	F: TCA ATG GAG TAA GCC CAA AG R: CAA GAG ACC GAG CAA TCA AG	59	28	246
*Tsg101*	F: ATG GCG GTG TCC GAG AGT CAG R: TTG ACA GTT TGA CGG ACG GT	55	33	80
*Ripk1*	F: GAA GAC AGA CCT AGA CAG CGG R: CCA GTA GCT TCA CCA CTC GAC	58	35	182
*Ripk3*	F: CAC ATA CTT TAC CCT TCA GA R: TCA GAA CAG TTG TTG AAG AC	58	35	172
*Mlkl*	F: GAC CAA ACT GAA GAC AAG TA R: CTC ACT ATT CCA ACA CTT TC	57	35	114
*Aqp8*	F: GGG GCA GCC TTT GCC ATC GT R: AAG AGG CCA GCC AGG AGG GG	59	28	296

### Examination of mice on days 4 and 6 of pregnancy

*Tsg101*^*f/f*^ and *Tsg101*^*d/d*^ female mice (9 to 13-week-old) received an intraperitoneal injection of 2.5 IU of eCG and hCG at 48 h intervals to promote mating. Immediately after hCG injection, females were bred with stud male mice. On the following morning, the formation of a vaginal plug was confirmed, and females with plugs were considered to be on day 1 of pregnancy. To examine implantation sites on day 6 of pregnancy, mice received a blue dye injection (1% Chicago blue B in phosphate buffered saline (PBS; Gibco, Thermo Fisher Scientific, Waltham, MA, USA) and sacrificed 3 min later. When no implantation site was visible, uteri were flushed with M2 medium (M7167, Sigma-Aldrich). Some mice were sacrificed at 11 AM on day 4 of pregnancy to confirm the presence of embryos. One uterine horn was flushed with M2 media and the other was processed for histological analyses.

### Pseudopregnancy

*Tsg101*^*f/f*^ and *Tsg101*^*d/d*^ female mice at 10 to 11-weeks of age received 2.5 IU of eCG and hCG at 48 h intervals. Immediately after hCG injection, the mice were bred with vasectomized ICR male mice. On the following morning, the formation of a vaginal plug was confirmed and females with plugs were considered to be on day 1 of pseudopregnancy. The uteri were collected from days 1, 4, or 6 of pseudopregnancy, and used for histological analysis and immunofluorescence staining. Uteri from day 4 pseudopregnant mice were used for uterine cell preparations.

### Histological analyses

The uteri from pregnant or pseudopregnant mice were cut into small pieces and fixed in 4% paraformaldehyde (PFA) in PBS overnight. Using a tissue processor, samples were dehydrated and embedded in paraffin. Sections (6–8 μm) were made using a microtome, placed onto a glass slide, and then subjected to hematoxylin-eosin (HE) staining. Slides were then examined using an upright microscope (Eclipse 80i, Nikon, Tokyo, Japan).

### Isolation of mouse uterine epithelial cells (UECs) and uterine stromal cells (USCs)

Uteri from random cycling ICR (8-week-old), *Tsg101*^*f/f*^, or *Tsg101*^*d/d*^ mice received a subcutaneous injection of E_2_ (100 ng/0.1 ml in sesame oil) 24 h before sacrifice to induce proliferation of UECs. Uteri pooled from 3 to 5 mice were cut into 3–4 mm pieces. Pancreatin (P3292; Sigma-Aldrich), dispase (17105–041; Gibco, Thermo Fisher Scientific), and collagenase (C1639; Sigma-Aldrich) were used to isolate uterine epithelial cells (UECs) and uterine stromal cells (USCs) as previously described [20]. Isolated UECs were filtered through a 70 μm nylon mesh filter (Corning, Sigma-Aldrich) to improve purity. UECs (2 × 10^5^ cells) were grown on collagen-coated coverslips in a 6-well plate (Corning, Sigma-Aldrich) in DMEM/F12 (Gibco, Thermo Fisher Scientific) supplemented with 10% fetal bovine serum (FBS) (Gibco, Thermo Fisher Scientific) and 1% penicillin-streptomycin (Lonza).

### Cell culture and necroptosis induction

The L929 fibroblast cell line derived from mouse adipose tissue was obtained from the Korean Cell Line Bank (Seoul, Korea). L929 cells were cultured in DMEM media (11965–092, Gibco, Thermo Fisher Scientific) supplemented with 10% FBS (10099–141, Gibco, Thermo Fisher Scientific) and 1% penicillin-streptomycin (17-602E, Lonza, Basel, Switzerland). To induce necroptosis, UECs were treated with a mixture of 30 ng/mL TNF-α (PeproTech, Rocky Hill, NJ, USA), 10 μM Smac mimetic LCL-161 (R&D Systems, Minneapolis, MN, USA), and 20 μM ZVAD-FMK (R&D Systems) for 40 min [13]. Control cells were treated with 0.2% dimethyl sulfoxide (vehicle). L929 cell lysates were used as positive controls in western blotting.

### RNA extraction and reverse transcription-polymerase chain reaction (RT-PCR)

Total RNA was extracted from pooled UECs and USCs isolated from several mice using the TRIzol Reagent (Invitrogen, Carlsbad, CA, USA) according to the manufacturer’s protocol. RNA was treated with RQ RNase-free DNase (Promega, Madison, WI, USA) to remove any genomic DNA for 20 min at 25 °C, followed by 10 min at 75 °C to inactivate the DNase. RNA concentration and quality were assessed using a NanoDrop (ND-1000; Thermo Fisher Scientific). Complementary DNA (cDNA) was synthesized from RNA using MMLV reverse transcriptase (BeamsBio, Seoul, Korea) and random hexamer primers (Invitrogen). Primers used for RT-PCR analyses are listed in Table 1. *Keratin 12 (Krt12)* and *desmin (Des)* were used as markers of the uterine epithelium and stroma, respectively [21].

### Western blotting

Isolated UECs and USCs were collected in RIPA buffer [10 mM Tris (pH 7.2), 150 mM NaCl, 0.1% Triton X-100, 5 mM ethylenediaminetetraacetic acid, 1% sodium dodecyl sulfate (SDS), 1 mM dithiothreitol, 1 mM phenylmethylsulfonyl fluoride, and 1X protease inhibitors) and homogenized. The lysates were centrifuged at 12,600×g for 15 min at 4 °C and the supernatants collected. A bicinchoninic acid protein assay (Thermo Fisher Scientific) was performed to determine the concentration of the extract. The lysates were prepared in 4X sample buffer and boiled for 5 min. Samples were loaded onto SDS-polyacrylamide gels and transferred onto polyvinylidene difluoride membranes (Millipore, Billerica, MA, USA). Membranes were blocked with 5% skim milk for 1 h and incubated overnight at 4 °C with the primary antibodies shown in Table 2. The membranes were washed three times and then incubated with secondary antibodies (Table 2) at 25 °C for 1 h. Chemiluminescence signals were detected using the West Save Detection Reagent A (Ab Frontier, Seoul, Korea) or West Femto kit (Thermo Fisher Scientific) and visualized with a LAS 4000 system (Fujifilm, Tokyo, Japan).

**Table 2:** Antibodies used in this study

Antibody	Host	Cat. no	Supplier	Dilution	Application
β-tubulin	Rabbit	ab6046	Abcam	1:2000	WB
RIPK1	Mouse	610,459	BD biosciences	1:500	WB/IF
pRIPK1	Rabbit	83,613	Cell signaling	1:500	WB
pRIPK1	Rabbit	31,122	Cell Signaling	1:1000	IF
RIPK3	Rabbit	NBP1–77299	Novus	1:500	WB/IF
pRIPK3	Rabbit	91,702	Cell signaling	1:500	WB
pRIPK3	Rabbit	57,220	Cell Signaling	1:1000	IF
MLKL	Rat	MABC604	Merk	1:500	WB/IF
pMLKL	Rabbit	ab196436	Abcam	1:500	WB/IF
E-cadherin	Rabbit	3195	Cell signaling	1:200	IF
EEA1	Rabbit	2411	Cell signaling	1:250	IF
Lamp1	Rat	NB100–77683	Novus	1:125	IF
Desmin	Goat	Sc7559	Santa Cruz	1:250	IF
Anti-rabbit IgG-HRP	Goat	SA002–500	GenDEPOT	1:10000	WB
Anti-mouse IgG-HRP	Goat	SA001–500	GenDEPOT	1:10000	WB
Anti-rat IgG-HRP	Goat	62–9520	Invitrogen	1:10000	WB
Anti-rabbit IgG-Alexa Fluor 488	Chick	A21441	Invitrogen	1:250	IF
Anti-rat IgG-Alexa Fluor 488	Donkey	A21208	Invitrogen	1:250	IF
Anti-goat IgG-Alexa Fluor 488	Rabbit	A21222	Invitrogen	1:250	IF
Anti-mouse IgG-Alexa Fluor 488	Donkey	A31571	Invitrogen	1:250	IF

### Immunofluorescence staining and confocal microscopy

Cells were fixed in 4% PFA for 10 min and washed three times with PBS for 3 min each. Cells were then permeabilized with 0.25% Triton X-100 for 10 min and blocked with 2% bovine serum albumin (BSA) in PBS for 1 h at 25 °C. The cells were incubated with primary antibodies overnight at 4 °C. After washing, the slides were incubated with secondary antibodies at 25 °C for 1 h in the dark. DNA was counter-stained with TOPRO-3-iodide (Invitrogen).

For immunofluorescence staining of uterine sections, pieces from the uteri of *Tsg101*^*f/f*^ and *Tsg101*^*d/d*^ mice were fixed in 4% PFA in PBS overnight, followed by 30% sucrose solution overnight. The tissues were then frozen in optimal cutting temperature compound (Leica Biosystems, Wetzlar, Germany) with instant freezing aerosol. Sections (12 μm) were made using a cryostat (Leica Biosystems). The frozen sections were fixed in 4% PFA and permeabilized with 0.1% Tween-20 at 25 °C for 20 min. After blocking with 2% BSA in PBS for 1 h at 25 °C, the sections were incubated with primary antibodies overnight at 4 °C. After washing, the slides were incubated with secondary antibodies at 25 °C for 1 h in the dark. DNA was counter-stained with TOPRO-3-iodide. Images were obtained with a Zeiss LSM900 confocal microscope (Carl Zeiss AG, Oberkochen, Germany) and analyzed with the ZEN Blue software (Carl Zeiss AG). Primary and secondary antibodies are shown in Table 2.

### TUNEL assay

Apoptosis was analyzed using the DeadEnd Fluorometric terminal deoxynucleotidyl transferase-mediated dUDP nick end labeling (TUNEL) assay kit (G3250; Promega). Paraffin sections of day 4 pseudopregnant uteri were deparaffinized in xylene, rehydrated through a graded series of ethanol, and washed with PBS. The sections were fixed in 4% PFA for 25 min and then permeabilized with 0.1% Triton X-100 for 5 min. The slides were equilibrated with equilibration buffer for 10 min and then incubated with recombinant terminal deoxynucleotidyl transferase incubation buffer at 37 °C for 1 h and covered with plastic coverslips. Sections were incubated with 2X saline sodium citrate buffer for 15 min and washed with PBS three times. The sections were counter-stained with TO-PRO-3-iodide (1:250 in PBS) for 15 min at 25 °C in the dark and rinsed three times in PBS for 5 min each. The slides were mounted in antifade reagent (Invitrogen), examined under a Zeiss LSM900 confocal microscope and analyzed with the ZEN Blue software.

### Live imaging during UEC culture

Isolated UECs were cultured in 60 mm dishes in culture medium. The cells were placed under a JuLI™ FL time-lapse microscope (JuLI-B004, NanoEntek, Seoul, Korea) in a CO_2_ incubator. For activation of necroptosis, TNF-α (30 ng/mL), Smac mimetic LCL161 (10 μM), and z-VAD (20 μM) were added to the culture media as described above. UECs were stained with SYTOX™ Green Nucleic Acid Stain (S7020, Invitrogen) and imaged automatically at 1 h intervals.

### RNA expression profiling

To compare the RNA expression profiles between *Tsg101*^*f/f*^ and *Tsg101*^*d/d*^ UECs, uteri from 3 *Tsg101*^*f/f*^ or 4 *Tsg101*^*d/d*^ mice were pooled and RNA extracted (*n* = 3 for each group). RNA quality was assessed using the 2100 Bioanalyzer system (Agilent Technologies, Santa Clara, CA, USA). Total RNA (1 μg) obtained from the samples was used for RNA extraction with the MGIEasy RNA Directional Library Prep Kit (LAS, Gimpo, Gyeonggi-do, Korea) and processed for high-throughput sequencing using MGISEQ-2000. Volcano plots for the expression-fold changes and *p*-values between the two selected samples were plotted by in-house R scripts. The top differentially expressed genes (DEGs) with ≥2-fold change (*p* ≤ 0.05) are shown as a heatmap, also drawn by an in-house R script. Significant changes in the biological processes based on Gene Ontology (GO), Kyoto Encyclopedia of Genes and Genomes (KEGG) pathways, and other functional gene sets were analyzed by g:Profiler version 0.6.7 [22].

### Statistical analysis

Data analysis and graph preparation were done using GraphPad Prism 5 software (https://www.graphpad.com/scientific-software/prism/) (Graph Pad Software, San Diego, CA, USA). For statistical analysis of RT-PCR, band intensities were measured using NIH ImageJ software and normalized to housekeeping gene expression. A Student’s *t*-test was conducted.

## Results

### Generation of uterine epithelium-specific *Tsg101* deletion model

**Fig. 1:**
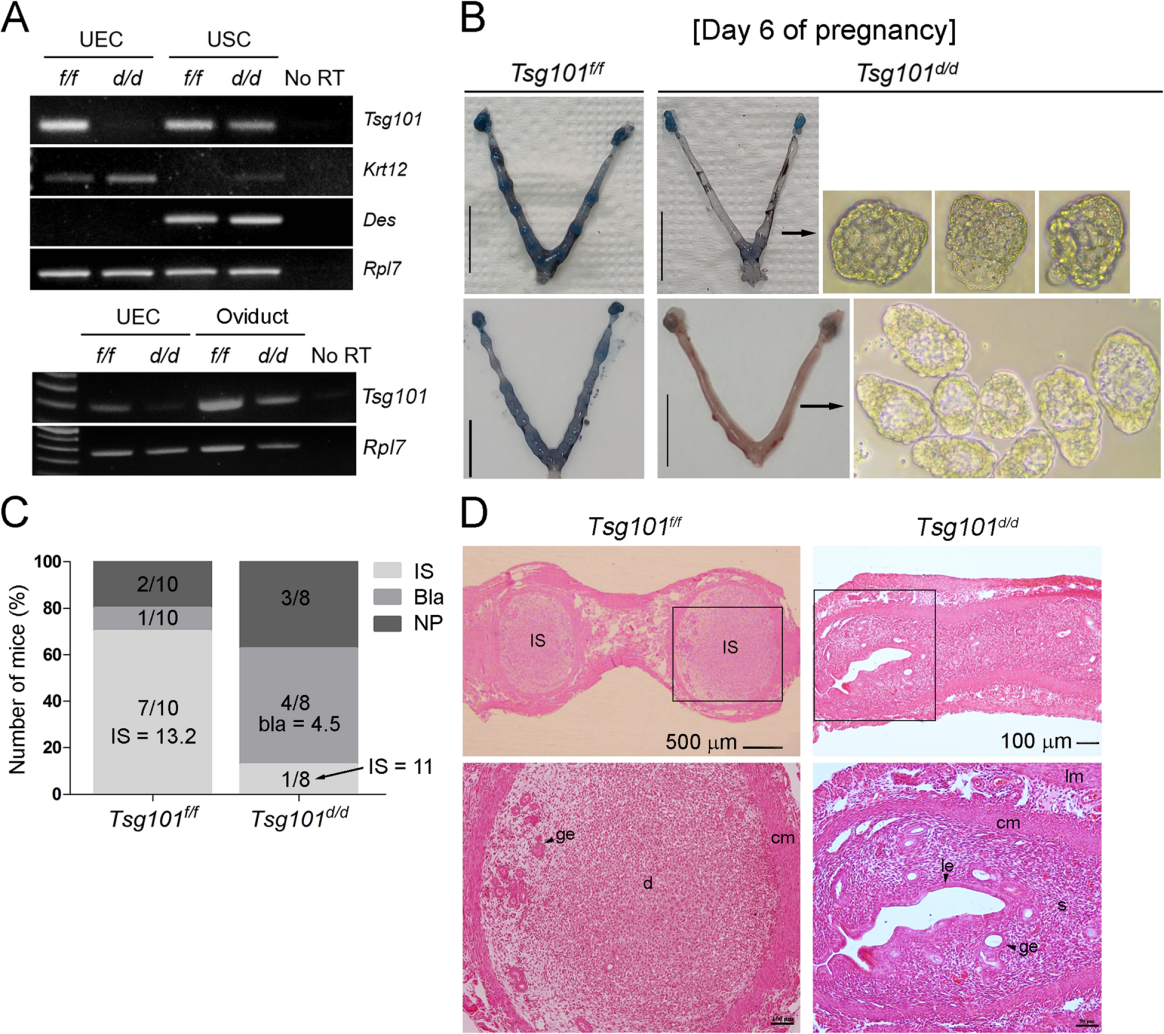
Compromised implantation in day 6 pregnant *Ltf-iCre/Tsg101*^*f/f*^ mice. (A) Uterine epithelium-specific deletion of floxed *Tsg101* gene by *Ltf-iCre* recombinase was confirmed by RT-PCR. Uterine epithelial cells (UECs) and uterine stromal cells (USCs) were isolated from 8 to 10-week-old random cycling *Tsg101*^*f/f*^ or *Ltf-iCre/Tsg101*^*f/f*^ (*Tsg101*^*d/d*^) mice. Deletion of *Tsg101* in *Tsg101*^*d/d*^ UECs was confirmed. *Keratin 12* (*Krt12*) and *desmin* (*Des*) were used as marker genes for UECs and USCs, respectively. *Ribosomal protein L7* (*Rpl7*) is a housekeeping gene and was used as an internal control. Five mice were used for cell isolation in each group. The bottom panel shows expression of *Tsg101* in *Tsg101*^*f/f*^ and *Tsg101*^*d/d*^ oviducts. (B) Uteri of day 6 pregnant *Tsg101*^*f/f*^ or *Tsg101*^*d/d*^ mice. Mice (10–11-week-old) received 2.5 IU of eCG and hCG and were bred with stud male mice. On day 6 of pregnancy, the mice received a blue dye injection to demarcate the implantation sites (IS). Uteri from mice without IS were flushed. Scale bar, 1 cm. (C) *Tsg101*^*f/f*^ (*n* = 10) or *Tsg101*^*d/d*^ (*n* = 8) with variable pregnancy outcomes on day 6. Most of the *Tsg101*^*f/f*^ mice had IS (average number = 13.3), whereas 50% of the *Tsg101*^*d/d*^ mice showed unimplanted blastocysts upon uterine flushing. IS, mice with implantation sites; Bla, mice with unimplanted blastocysts; NP, not pregnant. Average number of implantation sites or blastocysts is shown on the graph. (D) Histological analysis of day 6 pregnant uterine sections. A representative set of figures is shown. Paraffin-embedded sections were stained with hematoxylin and eosin. IS, implantation site; ge, glandular epithelium; cm, circular muscle; lm, longitudinal muscle; le, luminal epithelium; s, stroma. Areas demarcated with a black square are magnified in the lower panels

The uterine epithelium-specific deletion of Tsg101 was achieved by crossing *Tsg101*^*f/f*^ mice [10] with *Ltf-iCre* mice [19]. Deletion of *Tsg101* in isolated UECs, but not in the uterine stromal cells (USCs), was confirmed by RT-PCR (Fig. 1A).

### Implantation failure in *Tsg101*^*d/d*^ mice on day 6 of pregnancy

Three *Tsg101*^*d/d*^ female mice were bred with stud male mice for 8 months; however, only one of three gave birth to a small number of pups (2–4 pups, three times), suggesting compromised fertility. The status of pregnancy in the *Tsg101*^*d/d*^ mice was examined on day 6 of pregnancy when implantation sites (IS) are generally visible. As shown in Fig. 1B, 6 out of 7 pregnant *Tsg101*^*d/d*^ mice showed no IS, whereas control mice showed evenly spaced IS. The uterine flushings of *Tsg101*^*d/d*^ uteri (4 out of 8 mice), showed unimplanted, zona-free blastocysts (Fig. 1B & D). *Tsg101*^*d/d*^ uteri on day 6 showed variable thickness, as shown in Fig. 1B. Notably, the entire or portion of the uterus in some *Tsg101*^*d/d*^ mice showed fluid accumulation within the lumen, which seeped out during preparation (see Fig. 1B). Uterine histology of *Tsg101*^*d/d*^ mice showed that the overall uterine architecture was normal, with all major cell types and glands visible (Fig. 1D). However, no luminal closure or decidualization was observed on day 6 **(**Fig. 1D**)**, suggesting that the implantation reaction was not initiated.

### Delayed embryonic development in *Tsg101*^*d/d*^ uteri on day 4 of pregnancy

**Fig. 2:**
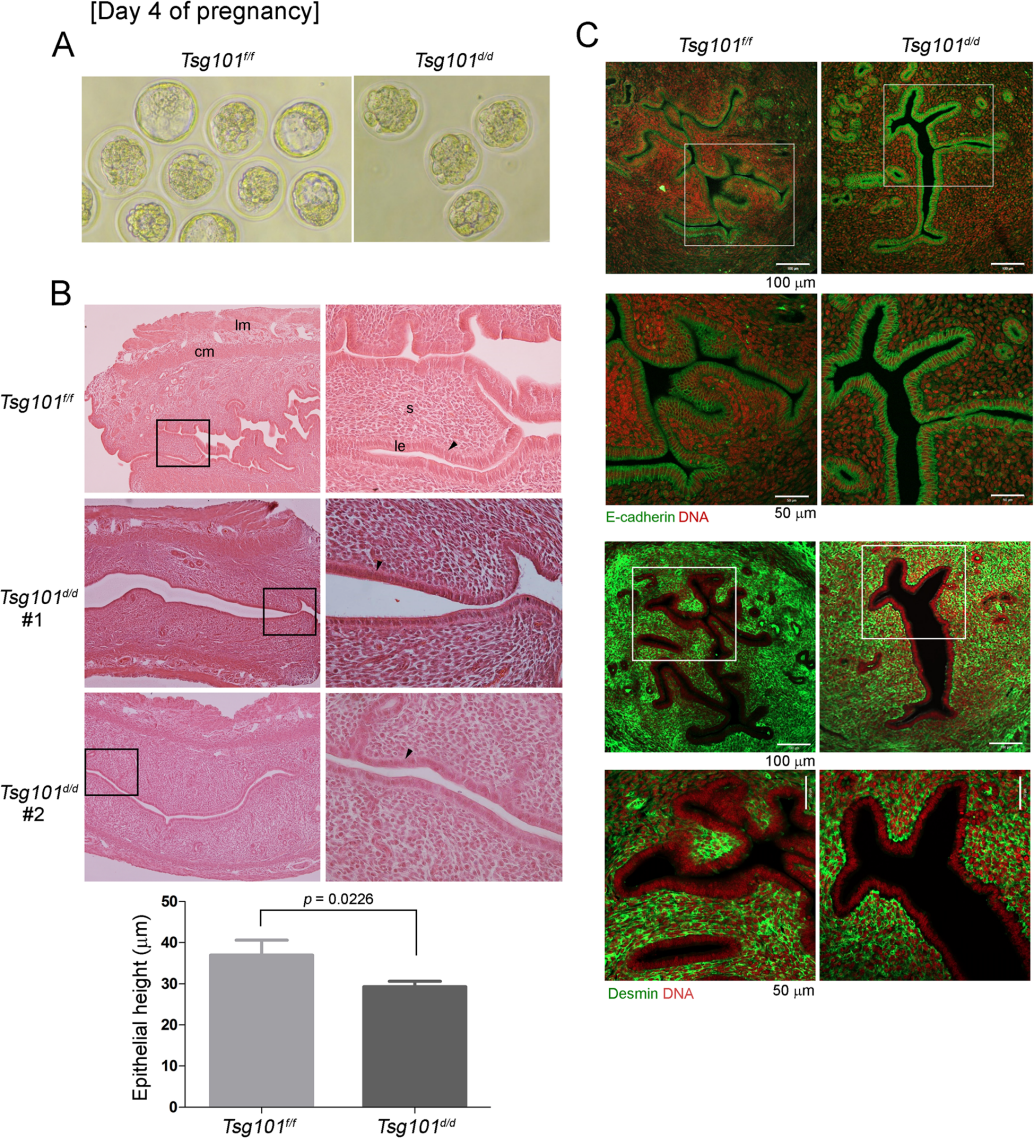
Day 4 pregnancy in *Tsg101*^*d/d*^ mice. (A) Mice (10–11-week-old) received 2.5 IU of eCG and hCG and were bred with stud male mice. On day 4 at 11 AM, the uteri were collected and one horn was flushed with warm M16 media. A set of representative images of the retrieved embryos are shown. See Table 3. (B) Histological analysis of day 4 pregnant uterine sections. Unflushed uterine horns were used for this experiment. Paraffin-embedded sections were stained with hematoxylin and eosin. lm, longitudinal muscle; cm, circular muscle; s, stroma; le, luminal epithelium. Areas demarcated with a black rectangle are magnified in the right panels. Sections from two different *Tsg101*^*d/d*^ uteri are shown as #1 and #2. Black arrowheads indicate the luminal epithelia. Two sections from different subjects were chosen and heights of the luminal epithelia were measured in several different areas. The measurement of epithelial heights is shown in graph. Bars represent means ± SEM. (C) Immunofluorescence staining of E-cadherin (epithelial cell marker) and desmin (stromal cell marker) was performed in one set of day 4 pregnant mice to show cell identity. Unflushed uterine horns were used for this experiment. Areas demarcated with a white square are magnified in the lower panels. DNA was counterstained with TO-PRO™-3-Iodide (1:250)

We next examined if embryonic development proceeds normally to the blastocyst stage by day 4 of pregnancy in *Tsg101*^*d/d*^ mice when the uterus is receptive to implantation. One uterine horn of *Tsg101*^*f/f*^ and *Tsg101*^*d/d*^ mice was flushed on day 4 of pregnancy, and the developmental stage of the embryos was monitored (Fig. 2, Table 3). At 11 AM on day 4, most embryos (81.25%) from *Tsg101*^*f/f*^ mice were at the blastocyst stage, whereas only 43.3% of the embryos from the *Tsg101*^*d/d*^ uteri were at the same stage (Table 3). These results show that embryonic development in *Tsg101*^*d/d*^ mice is marginally delayed. Nonetheless, the presence of blastocysts on day 4 in *Tsg101*^*d/d*^ mice, which failed to implant by day 6 of pregnancy was confirmed (Fig. 1B).

**Table 3:** The number of embryos in uterine flushings of day 4 pregnant mice

Genotype	No. of plug-positive mice	No. of mice with embryos	Total no. of embryos*	Total no. of blastocysts (%)	Total no. of morula (%)
*Tsg101*^*f/f*^	8	6	32	26 (81.25)	6 (18.75)
*Tsg101*^*d/d*^	9	6	30	13 (43.3)	17 (56.7)

* Mice received 2.5 IU of eCG followed by 2.5 IU hCG 48 h later and were individually caged with stud males to induce mating. The next morning, a mouse with a visible vaginal plug was designated to be on day 1 of pregnancy. Mice were sacrificed at 11 AM on day 4. One uterine horn from each mouse was flushed and the other horn was subjected to histological analysis

The unflushed uterine horn was subjected to histological analysis (Fig. 2B). The overall uterine structures in *Tsg101*^*d/d*^ mice appeared normal, but the luminal epithelium seemed shorter (Fig. 2B, arrowheads and graph). Overall histological analyses suggested that the luminal epithelia of *Tsg101*^*d/d*^ uteri on day 4 of pregnancy was less developed than those of *Tsg101*^*f/f*^ uteri, displaying shortening of apical lengths. The average height of the luminal epithelium in *Tsg101*^*d/d*^ uteri was approximately 30 μm, about 20% lower than that of the *Tsg101*^*f/f*^ uteri (average 37 μm). This observation suggests that epithelial differentiation, which occurs during preparation for implantation, requires Tsg101 for structural integrity. E-cadherin and desmin, markers of the epithelium and stroma, respectively, showed an expected pattern of localization in *Tsg101*^*d/d*^ uteri, with E-cadherin in the uterine epithelium and desmin in the stroma (Fig. 2C).

### Cultured *Tsg101*^*d/d*^ UECs show a high rate of degeneration

**Fig. 3:**
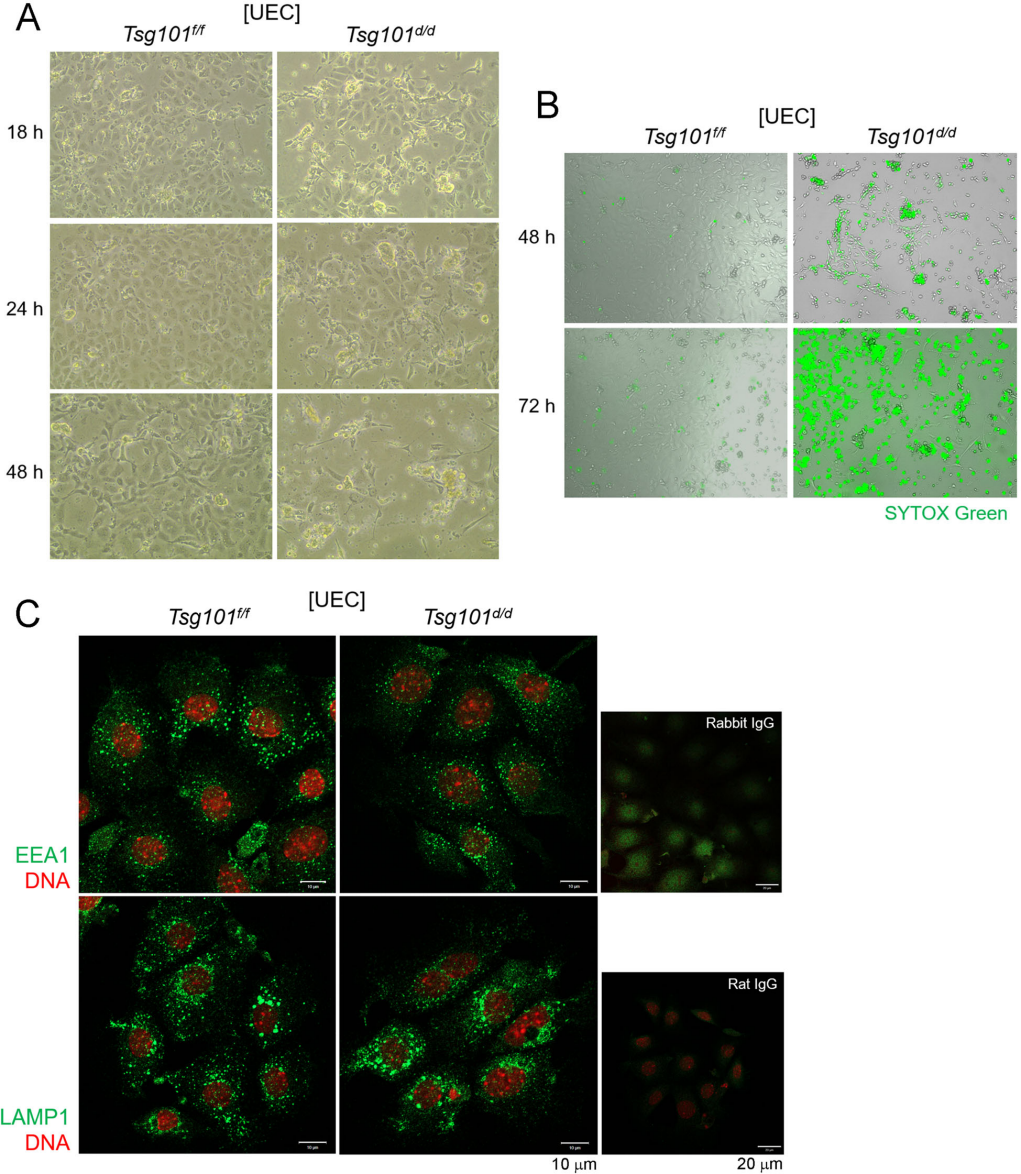
*Tsg101*^*d/d*^ UECs gradually degenerate during in vitro culture. (A) UECs were isolated from uteri pooled from 2 to 4 *Tsg101*^*f/f*^ and *Tsg101*^*d/d*^ mice (11-week-old) and placed in culture at 2 × 10^5^ cells per well. An injection of E_2_ was administered to the mice 24 h before sacrifice to increase the cell yield. The morphology of the cultured UECs was examined at the indicated times. Experiments were repeated four times with similar results. (B) Live cell imaging of *Tsg101*^*f/f*^ and *Tsg101*^*d/d*^ UECs by using JuLI™ FL. 48 h in culture, cells were stained with SYTOX Green, a live dye which stains DNA of membrane-permeable cells (cells with weakened membrane or dead cells). Experiments were repeated twice with similar results. (C) Immunofluorescence staining of EEA1 and LAMP1 in *Tsg101*^*f/f*^ and *Tsg101*^*d/d*^ UECs. UECs isolated from *Tsg101*^*f/f*^ and *Tsg101*^*d/d*^ mice (9-week-old) were cultured and subjected to immunofluorescence staining 18 h later. Cells were stained with indicated primary antibodies (green). DNA was stained with TO-PRO™-3-Iodine (1:250). Experiments were repeated three times with similar results

It has been previously shown that MEFs [10] and primary mammary epithelial cells [8] with *Tsg101* knockdown show poor survival and various subcellular abnormalities. Thus, UECs isolated from *Tsg101*^*d/d*^ uteri were cultured in vitro and cell survival was monitored. We observed that *Tsg101*^*d/d*^ UECs began to degenerate within 18 h of culture (Fig. 3A). After 72 h, the number of remaining *Tsg101*^*d/d*^ cells was much lower than *Tsg101*^*f/f*^ UECs and stained positive for SYTOX Green stain, which stains cells with compromised plasma membranes (Fig. 3B).

**Fig. 4:**
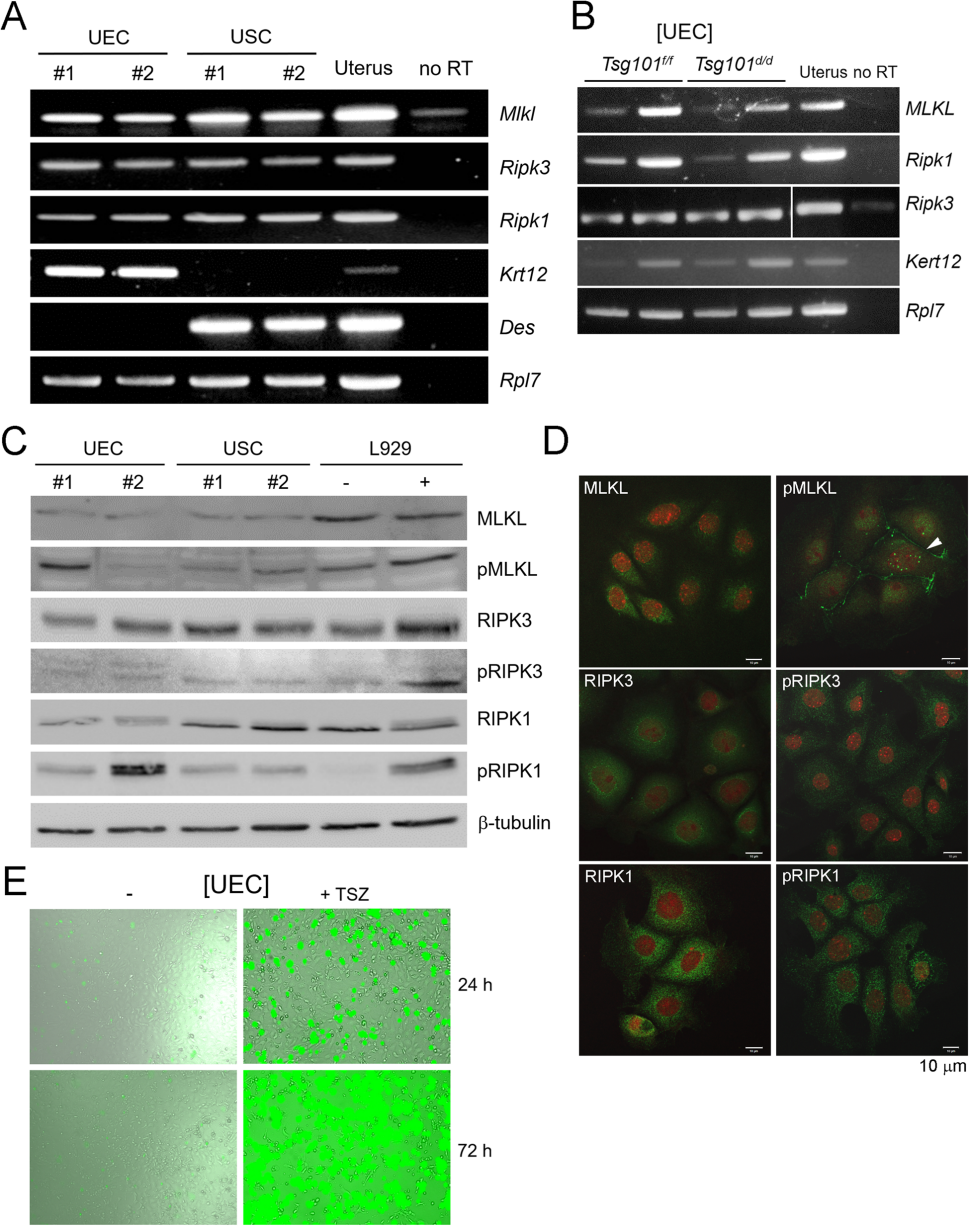
UECs express necroptosis effectors, RIPK1, RIPK3, and MLKL, and respond to necroptosis-inducing signal. (A) RT-PCR of necroptosis factors performed in isolated UECs, USCs, and uteri from random cycling ICR mice. Five mice were pooled for each group. E_2_ was given to mice 24 h before sacrifice to increase yield of UECs. Results from two sets of independent samples experiments are shown as #1 and #2. RNA from whole uteri was used as a positive control. *Mlkl*, *Mixed lineage kinase domain-like*; *Ripk3*, *Receptor interacting protein kinase 3*; *Ripk1*, *Receptor interacting protein kinase 1*; *Des*, *Desmin* (a stromal marker); *Krt12*, *Keratin 12* (an epithelial marker); *Rpl7*, *Ribosomal protein L7* (a housekeeping gene). Experiments were repeated three times with similar results. (B) RT-PCR of necroptosis effectors, Mlkl, Ripk1, and Ripk3 in UECs isolated from *Tsg101*^*f/f*^ and *Tsg101*^*d/d*^ mice. Two independent samples were used. *Mlkl*, *Mixed lineage kinase domain-like*; *Ripk3*, *Receptor interacting protein kinase 3*; *Ripk1*, *Receptor interacting protein kinase 1*; *Des*, *Desmin* (a stromal marker); *Krt12*, *Keratin 12* (an epithelial marker). (C) Western blot analyses of necroptosis effectors in UECs and USCs. L929 cells treated with TSZ were used as a positive control. pMLKL, phospho-MLKL; pRIPK1, phospho-RIPK1; pRIPK3, phospho-RIPK3. Two independent samples were used (#1 and #2), and experiments were repeated two times with similar results. (D) Immunofluorescence staining of necroptosis effects in cultured UECs. DNA was stained with TO-PRO™-3-Iodine (1:250). Experiments were repeated three times; a set of representative images are shown. (E) Isolated UECs were plated and treated with TSZ (TNFα + Smac mimetic LCL161 + zVAD-fmk) or DMSO (vehicle) the day after all cells had attached. TSZ was added at 24 h in culture along with SYTOX Green dye, which stains dead cells only. Live images were captured at 1 h interval using the JuLI^FM^ FL. TNFα, 30 ng/ml; Smac mimetic LCL161, 10 μM; zVAD-fmk, 20 μM

The main function of Tsg101 is correlated with cytokinesis, endosomal trafficking, and the formation of the late endosomal structures called MVBs [23]. Therefore, we examined whether endolysosomal structures in *Tsg101*^*d/d*^ UECs were normal. Localization of early endosome antigen 1 (EEA1) and lysosome-associated membrane protein 1 (LAMP1) was examined by immunofluorescence staining (Fig. 3C). As shown in Fig. 4C, both EEA1 and LAMP1 exhibited puncta-like localization mostly in the perinuclear region, which did not differ between the *Tsg101*^*f/f*^ and *Tsg101*^*d/d*^ UECs. These results collectively suggest that cultured UECs tend to degenerate in the absence of Tsg101 without noticeable endolysosomal defects. Thus, increased UEC death could be associated with implantation failure in *Tsg101*^*d/d*^ mice.

### Expression of necroptosis factors in UECs

Another role for Tsg101 and other ESCRT factors has recently been suggested which involves counteracting necroptotic cell death [11]. Necroptosis can be induced by various external and internal stimuli, and the resulting plasma membrane breach is generally mediated by the RIPK1-RIPK3-MLKL pathway [24]. Whether UECs express such necroptosis effectors has not been reported. We first confirmed the expression of *Ripk1*, *Ripk3*, and *Mlkl* in isolated UECs and USCs (Fig. 4A). *Tsg101*^*d/d*^ UECs also expressed these factors (Fig. 4B).

Using TSZ-treated L929 cells as a positive control, we examined the status of RIPK1, RIPK3, MLKL, and their phosphorylated forms by western blotting. As shown in Fig. 4C, all three factors were present in both UECs and USCs. Since their phosphorylated forms were also detected in UECs and USCs without external stimulation, it is possible that a basal level of necroptosis may be operative in these cells. Immunofluorescence staining of these factors mostly showed a scattered punctate pattern in the cytoplasm. As for phosphorylated MLKL (pMLKL), it was localized in some UECs in the plasma membrane, which is known to occur upon activation of necroptosis (Fig. 4D, white arrowhead) [25].

We then tested whether UECs respond to exogenous necroptosis-inducing signals. UECs were treated with TSZ for 24 h in the presence of the SYTOX Green live dye. As shown in Fig. 4E, TSZ treatment dramatically increased SYTOX Green-positive UEC cells. Thus, UECs are equipped with necroptosis effectors and can respond to necroptosis-inducing exogenous signals.

### Cell death in *Tsg101*^*d/d*^ UECs

**Fig. 5:**
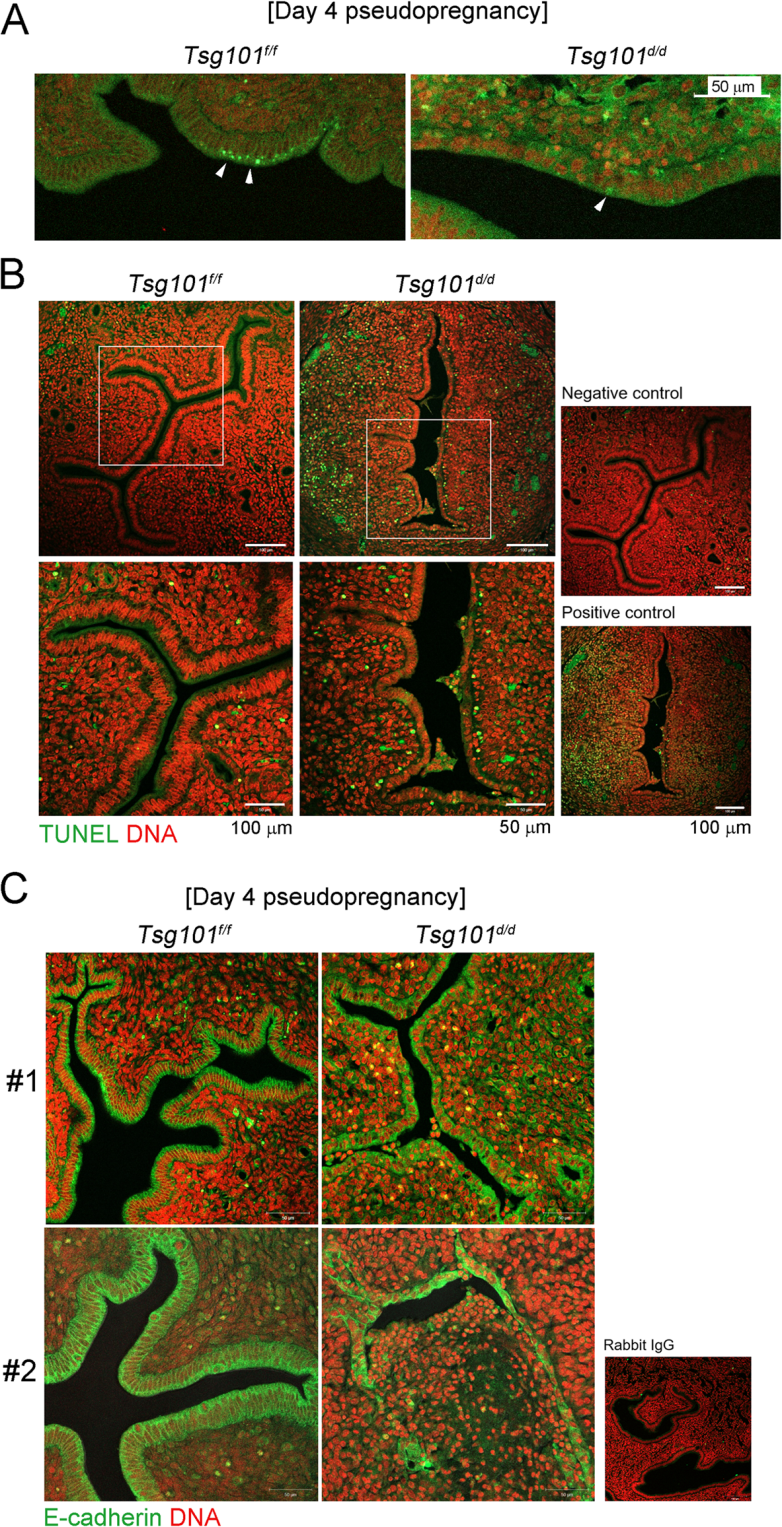
pMLKL and E-cadherin immunofluorescence staining and TUNEL assay in day 4 pseudopregnant *Tsg101*^*d/d*^ uteri. (A) Immunofluorescence staining of pMLKL in day 4 pseudopregnant uteri (*n* = 2 each). One representative set is shown. (B) TUNEL staining in day 4 pseudopregnant uteri to observe apoptotic cells (n = 2 each). Green, apoptotic cell; red, nuclei. Areas demarcated with white rectangles are enlarged in the lower panel. One representative set is shown. (C) E-cadherin localization on day 4 of pseudopregnancy. Two independent samples are shown as #1 and #2 (*n* = 3). Uterus #2 showed the most severe phenotype of epithelial disintegration, whereas #1 showed shortened luminal epithelial height

The final executor of necroptosis is pMLKL, which induces permeabilization of the plasma membrane [12]. Whether the luminal epithelium expresses active pMLKL during pregnancy is unknown. We examined whether pMLKL is localized to the luminal epithelium on day 4 of pseudopregnancy (Fig. 5A). In the *Tsg101*^*f/f*^ uteri, pMLKL showed a punctate localization in a portion of the apical surface of the luminal epithelium (Fig. 5A). In the *Tsg101*^*d/d*^ uteri with shortened luminal epithelium, the pMLKL signal was not as distinct as in the *Tsg101*^*f/f*^ uteri.

To investigate whether detachment of cells in the *Tsg101*^*d/d*^ uterus is associated with apoptosis, we performed TUNEL staining on day 4 pseudopregnant uterine sections (Fig. 5B). We found cells detached from the luminal epithelium and a higher number of TUNEL-positive cells in the subepithelial stromal regions in the *Tsg101*^*d/d*^ uterus.

During the examination of the epithelial morphology in pseudopregnant *Tsg101*^*d/d*^ uteri using E-cadherin as a marker, we noticed that in one *Tsg101*^*d/d*^ uterus the luminal epithelium had disintegrated. As shown in Fig. 5C, E-cadherin was localized to the uterine epithelium in both groups, but *Tsg101*^*d/d*^ uteri showed abnormalities. One (#1) *Tsg101*^*d/d*^ uterus showed an epithelial mass detached from the luminal epithelium, whereas the other (#2) uterus showed a disintegrated and collapsed luminal epithelium (Fig. 3B). We were able to distinguish the epithelial tissue, because the cells retained E-cadherin localization. Thus, it seems that *Tsg101*^*d/d*^ uterine epithelium retained its epithelial characteristics with intact marker expression but partially lost structural integrity. These results suggest that implantation failure in *Tsg101*^*d/d*^ mice is associated with epithelial defects in the absence of *Tsg101*.

### mRNA expression landscape in the *Tsg101*^*f/f*^ and *Tsg101*^*d/d*^ UECs

**Fig. 6:**
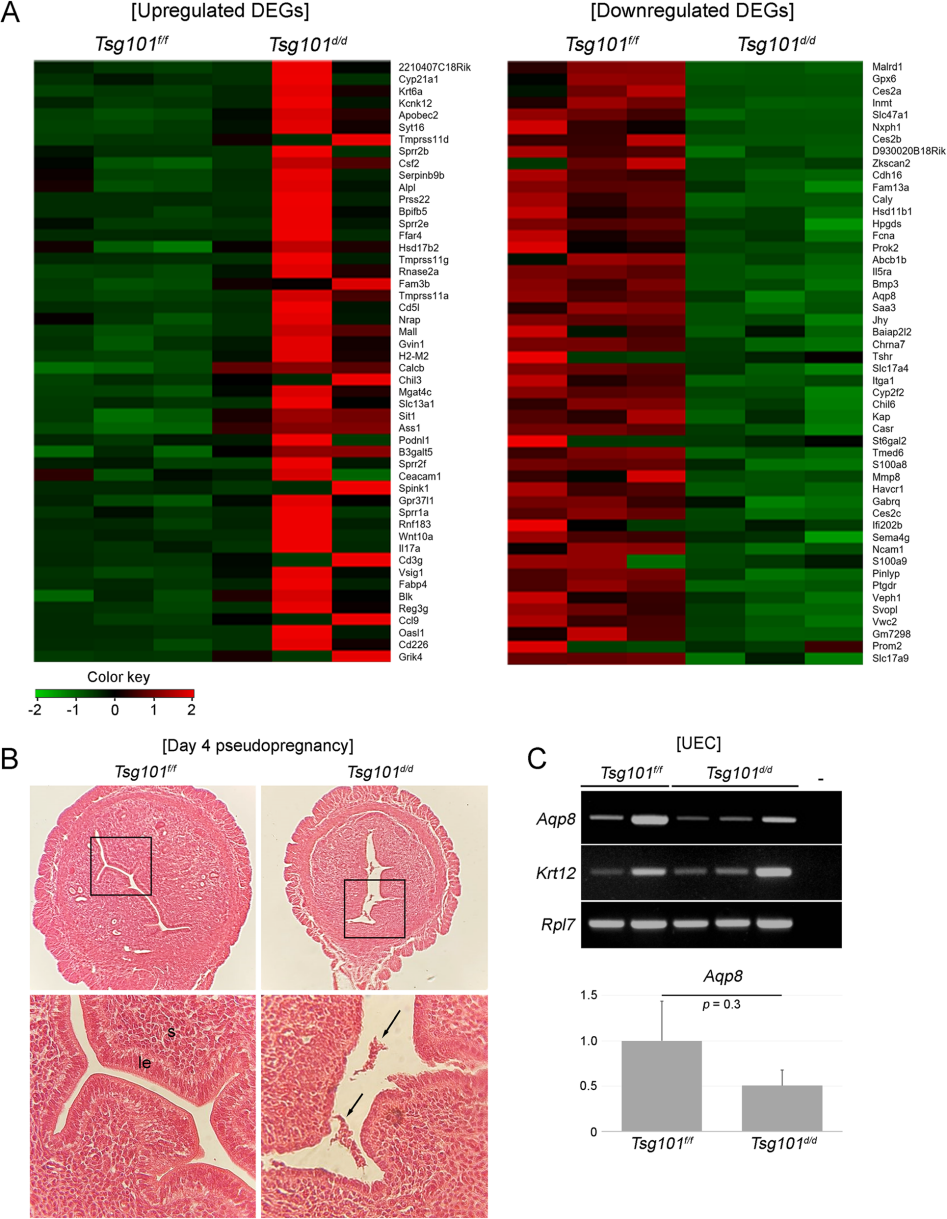
mRNA expression profiling in UECs from *Tsg101*^*f/f*^ and *Tsg101*^*d/d*^ mice. (A) Top 50 upregulated and downregulated genes are presented as heatmaps. For each sample, 3–5 mice were pooled. Three sets were prepared and shown in the figure (B) A representative histological image of day 4 pseudopregnant uteri used for mRNA expression profiling. Arrows indicate detached epithelial tissues in a *Tsg101*^*d/d*^ uterus. le, luminal epithelium; s, stroma. Experiments were repeated two times with similar results. One representative set is shown. (C) RT-PCR analyses of *Aqp8* in UEC RNA samples. Two *Tsg101*^*f/f*^ UEC and three *Tsg101*^*d/d*^ UEC samples were used. -, no RT. The gene expression of Aqp8 was normalized with *Rpl7* levels. No significant difference between samples was observed

To compare the overall mRNA expression landscape between the *Tsg101*^*f/f*^ and *Tsg101*^*d/d*^ UECs, we performed mRNA expression profiling. To avoid mRNA contamination from the embryos, UECs from day 4 pseudopregnant mice were used. Pseudopregnancy models are widely used for uterine functions, but it is to be noted that uterine cells are not exposed to developing embryos. These samples were subjected to mRNA expression profiling. Heatmaps of the top 50 differentially expressed genes (DEGs) are shown in Fig. 6A. Remarkably, genes upregulated in the *Tsg101*^*d/d*^ UECs exhibited high variation between the samples (Fig. 6A, left panel), whereas genes downregulated in *Tsg101*^*d/d*^ UECs showed a more consistent trend (Fig. 6A, right panel). In total, 1284 genes were differentially expressed between *Tsg101*^*f/f*^ *and Tsg101*^*d/d*^ UECs. Of these DEGs, 734 genes were upregulated, whereas 550 genes were downregulated in *Tsg101*^*d/d*^. Histological examination of day 4 pseudopregnant uteri from *Tsg101*^*d/d*^ mice used in this experiment showed patches of cells in the lumen (Fig. 6B). Expression of *aquaporin 8* *(Aqp8*), one of the downregulated genes in *Tsg101*^*d/d*^ UEC, was examined. Although Aqp8 expression was low in *Tsg101*^*d/d*^ UEC, there was no statistical significance in this set of samples (Fig. 6C).

**Fig. 7:**
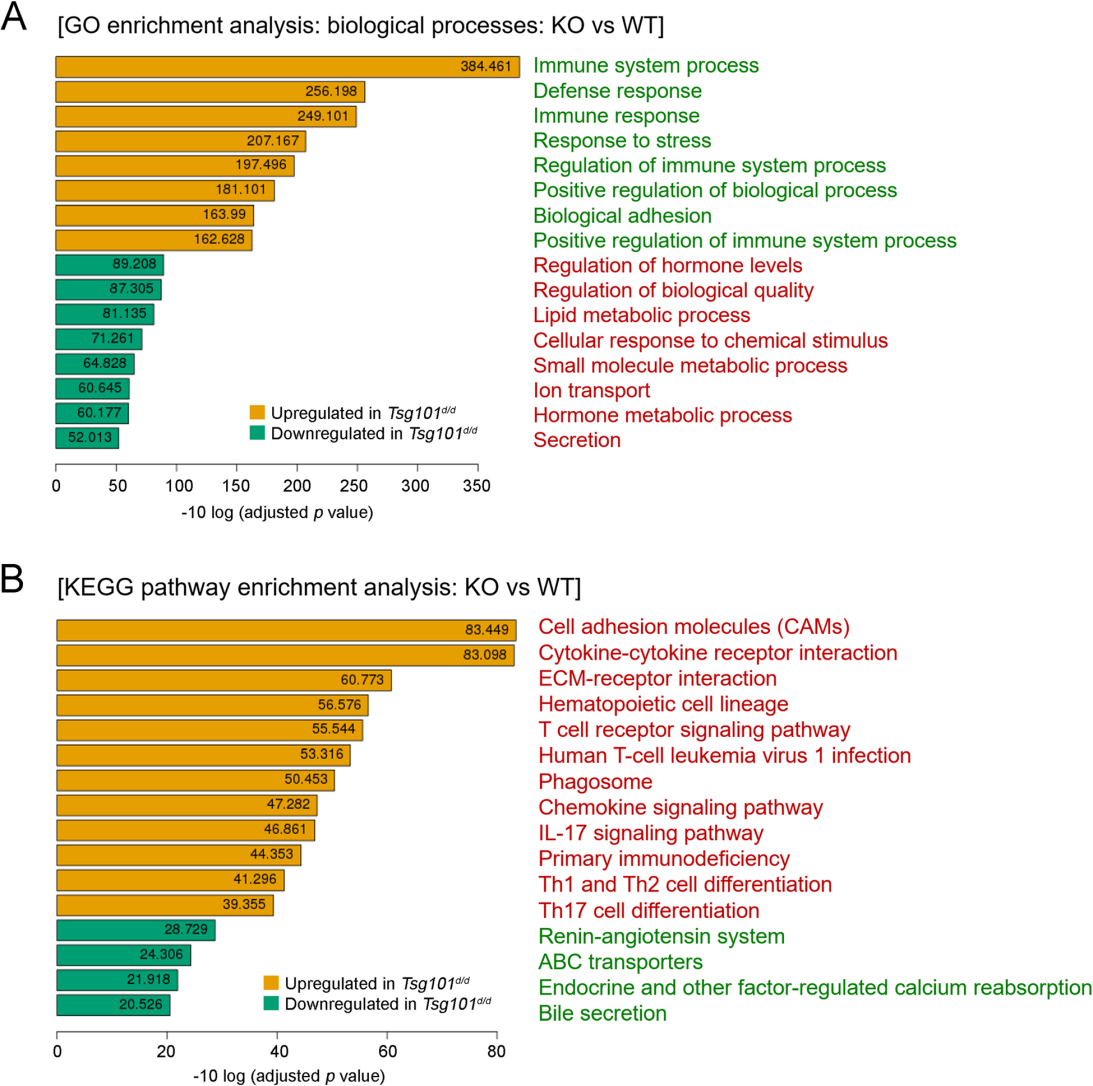
Pathway analyses of gene expression profiles. (A) Gene Ontology (GO) term enrichment analysis of biological processes for upregulated and downregulated DEGs between the *Tsg101*^*f/f*^ and *Tsg101*^*d/d*^ UECs. Top 16 GO terms associated with the biological processes are shown. The X-axis corresponds to the mean expression value of negative log 10 (adjusted *p* value). (B) Kyoto Encyclopedia of Genes and Genomes (KEGG) pathway enrichment analysis for the upregulated and downregulated genes between the *Tsg101*^*f/f*^ and *Tsg101*^*d/d*^ UECs. Gene expression information was mapped to the KEGG pathway and the top 12 significantly upregulated and the top 4 downregulated pathways are shown. The X-axis corresponds to the mean expression value of negative log 10 (adjusted *p* value)

GO term enrichment and KEGG pathway analyses were used to identify key genes and pathways operative in *Tsg101*^*f/f*^ and *Tsg101*^*d/d*^ UECs (Fig. 7). In terms of DEGs in GO term enrichment analysis for the biological process (Fig. 7A), several gene classes associated with immune functions were upregulated in *Tsg101*^*d/d*^ UECs. KEGG pathway analysis of the DEGs showed clustering of several signaling pathways, such as cell adhesion molecules and cytokine-cytokine receptor interaction (Fig. 7B). Together, these results suggest that various cellular functions were affected in the uterine epithelium in the absence of *Tsg101*.

## Discussion

*Tsg101* was initially cloned as a candidate tumor suppressor gene in mice [26]. While several reports suggest a role for this gene in tumor suppression [6], other complex and fundamental roles for Tsg101 in cells have been uncovered, ranging from endolysosomal maturation, cytokinesis, cell proliferation, and survival [6]. As the deletion of *Tsg101* in mice is lethal early in development [10], the biological functions of Tsg101 have been investigated in several tissue-specific *Tsg101* deletion mouse models [6]. In mammary epithelial cells, cardiomyocytes, and oligodendroglia, *Tsg101* deletion leads to cell death accompanied by apoptosis, vacuolation, and other subcellular changes [7, 27, 28].

Our study shows that Tsg101 plays a crucial role in maintaining the integrity of the uterine epithelium during early pregnancy. The *Ltf-iCre* mice achieved efficient and specific deletion of the floxed genes in the uterine epithelium at approximately 2 months of age [19]. All mice used in our experiments were between 8 and 12-weeks of age. By this time, all uterine structures have formed and sexual maturation is complete. Thus, the subfertility phenotype observed in *Tsg101*^*d/d*^ mice is irrelevant to anatomical and endocrinological abnormalities. Our results show that *Tsg101* is required in the uterine epithelium to initiate embryo implantation (Fig. 1). The presence of well-formed, zona-free blastocysts in day 6 pregnant *Tsg101*^*d/d*^ uteri suggests that the luminal epithelium is unable to support implantation.

On day 4 of pregnancy, *Tsg101*^*d/d*^ uteri contained preimplantation embryos at the morula and blastocyst stages (Fig. 2A). Since *Tsg101* deletion was achieved in the epithelium of the oviduct and uterus, *Tsg101* may be associated with the loss of certain epithelial factors during preimplantation embryonic development, leading to delayed embryonic development on day 4 (Table 3). This assumption is plausible considering the previous report that epidermal growth factor signaling is downregulated in Tsg101-depleted MEF cells [8]. The day 4 pregnant uterus is under the influence of progesterone and estrogen, both of which influence dynamic cellular and molecular changes required for implantation [29]. Among day 4 pseudopregnant *Tsg101*^*d/d*^ mice used in E-cadherin localization experiments, one mouse between 11 and 12-weeks of age showed the most severe phenotype of collapsed epithelial structure (Fig. 5C). Another uterine section of a day 4 pseudopregnant *Tsg101*^*d/d*^ mouse showed detached epithelial tissue in the lumen (Fig. 6B). This phenotype was quite challenging in terms of investigating gene expression profiles (Fig. 6A), as several *Tsg101*^*d/d*^ UEC samples did not show comparable levels of *Krt12* to *Tsg101*^*f/f*^ UEC samples and thus could not be included in the experiments (data not shown). Among at least 6 *Tsg101*^*d/d*^ UEC samples, we chose samples with sufficient amount of RNA and *Krt12* expression.

When UECs were isolated and cultured in vitro, *Tsg101*^*d/d*^ UECs began to show signs of degeneration around 24 h with the emergence of clustered cell patches (Fig. 3), which are uncharacteristic of epithelial cells. *Tsg101*^*d/d*^ UECs also showed increased cell permeabilization, as was previously observed in certain ESCRT factor-depleted cells [11]. It was previously shown that *Tsg101*-depleted MEFs showed enlarged lysosomal structures, along with other complex cellular changes [8]. In the UECs, we did not observe a similar pattern. In vertebrate epithelial cells, ESCRT factors have been implicated in the maintenance of polarity [9]. Considering that MEFs are of mesenchymal origin, Tsg101 and other ESCRT factors may play distinct roles depending on the cell type.

Necroptosis can be initiated by various stimuli, such as death ligands and bacterial toxins, but can also be induced during normal physiological conditions and aging [12, 30]. Here, we show for the first time that UECs express the major necroptosis effectors, RIPK1, RIPK3, and MLKL, and their phosphorylated forms (Fig. 4D, arrowhead). pMLKL localization to the cell edge (Fig. 5C) suggests that UECs show active necroptosis [11, 31]. When TSZ was used to induce necroptosis [13], UECs showed a dramatic increase in SYTOX Green staining, which further supports the notion that UECs respond to external stimuli and activate necroptosis. Consistent with this finding, the *Tsg101*^*f/f*^ uterine epithelium on day 4 of pregnancy showed a distinct punctate pattern of pMLKL localization on the epithelial edge (Fig. 5A). The presence of pMLKL implies active necroptosis involving the cell membrane. Thus, our results suggest that UECs harbor a functional necroptotic machinery. The degeneration of cultured *Tsg101*^*d/d*^ UECs and disintegration of the uterine epithelium in *Tsg101*^*d/d*^ uteri may be associated with a failure to counteract the necroptotic activation that occurs as a part of the normal physiology of these cells.

Finally, we compared RNA expression profiles between the *Tsg101*^*f/f*^ and *Tsg101*^*d/d*^ UECs (Fig. 6), but the RNA expression among the different *Tsg101*^*d/d*^ UEC samples tended to show a high variation. This may be associated with the structural disintegration of the luminal epithelium observed in some *Tsg101*^*d/d*^ uteri (Fig. 5C). Such high variation precluded us from pinpointing target pathways and genes associated with Tsg101 in the uterine epithelium. Since fluid accumulation within the lumen was observed in several *Tsg101*^*d/d*^ uteri (Fig. 1B), further investigation is warranted to examine whether dysregulation of water channels, including *Aqp8*, is associated with this phenotype [32]. This could be partially due to epithelial disintegration in some *Tsg101*^*d/d*^ mice (Fig. 3B), highlighting the importance of Tsg101 in maintaining uterine tissue architecture.

## Conclusions

To date, the role of necroptosis and ESCRT factors in regulating uterine physiology and embryo implantation is not known. We confirm, for the first time, the presence of necroptosis effectors in UECs. UECs also responded to an exogenous necroptosis-inducing stimulus, involving a combination of TNFα, Smac mimetics, and an apoptosis inhibitor, and showed increased membrane permeabilization. However, *Tsg101*^*d/d*^ UECs degenerated in vitro, even in the absence of such external stimuli. Thus, it is reasonable to assume that Tsg101 is required to sustain the survival of cultured UECs. Since UECs showed a tendency to disintegrate within *Tsg101*^*d/d*^ uteri in vivo, it would be interesting to investigate how the tissue architecture of the uterus is maintained in older *Tsg101*^*d/d*^ mice. Whether the uterine epithelium degenerates completely or cells of a different origin replace the epithelium in the *Tsg101*^*d/d*^ uteri, requires further investigation. Our model can be further applied to study cell-to-cell interactions during uterine regeneration. The regulation of necroptosis in UECs and its role in uterine physiology warrants further investigation. As there is no information currently on the expression of Tsg101 and necroptosis factors in the uterus of other species, including humans, this work will serve as a reference. The results of pathway analyses can further be applied to other epithelial systems in vitro to elucidate the mechanistic aspects of Tsg101 function. How this cell death mechanism is related to inflammation-associated uterine pathology is another relevant topic that needs to be pursued in the future.

## Data Availability

Data supporting the findings are presented within the manuscript.

## Abbreviations

ESCRT: Endosomal sorting complex required for transport
Tsg101: Tumor susceptibility gene 101
eCG: equine chorionic gonadotropin
hCG: human chorionic gonadotropin
MLKL: Mixed lineage kinase-like protein
RIPK1: Receptor interacting protein kinase 1
RIPK3: Receptor interacting protein kinase 3
